# The proline TP53 variant stimulates likely lymphangiogenesis in an orthotopic mouse model of pancreatic cancer

**DOI:** 10.1038/bjc.2011.521

**Published:** 2011-12-06

**Authors:** N Otto, P Schulz, A Scholz, P Hauff, B Schlegelberger, K M Detjen, B Wiedenmann

**Affiliations:** 1 Department of Internal Medicine, Division of Hepatology and Gastroenterology, Charité Berlin, Campus Virchow Clinic, 13353 Berlin, Germany; 2 Global Drug Discovery, Bayer Schering Pharma AG, 13353 Berlin, Germany; 3 Institute of Cell and Molecular Pathology, Hannover Medical School, Carl-Neuberg-Str. 1, 30625 Hannover, Germany

**Keywords:** TP53, pancreatic cancer, orthotopic model, lymphangiogenesis, metastasis

## Abstract

**Background:**

Pancreatic cancer is a deadly disease characterised by high incidence of *TP53* mutations. Restoration of TP53 function is perceived as a highly attractive therapeutic strategy, whose effects are not well characterised.

**Methods:**

The current work adapted an inducible strategy of stage-specific reexpression of wild-type (wt) TP53 in an *in vivo* orthotopic mouse model of pancreatic cancer.

**Results:**

The reconstitution of wt TP53 function in *TP53*-mutant DanG and MiaPaCa-2 cells caused G1 cell cycle arrest but no evidence of apoptosis induction. Consistent with subcutaneous xenograft models, we found that wt TP53 reduced primary tumour growth. Wt TP53 reexpression during early tumour growth led to significant increase in vascularisation. This correlated with an unexpectedly high rate of micro-metastases in lymph nodes of animals with wt TP53 induction, despite the 90% decrease in median primary tumour weight. Reexpression of wt TP53 later in tumour development did not significantly affect the number of CD31-reactive vessels, but increased lymphatic vessel density.

**Conclusion:**

The increased number of lymphatic vessels and micro-metastases suggests that wt TP53 induction complexly affected the biology of different tumour constituents of pancreatic cancer. Our observation suggests that combination of the inducible system with an orthotopic model can yield important insights into *in vivo* pancreatic cancer biology.

Human ductal pancreatic cancer is one of the most aggressive malignancies with a poor prognosis (Jemal *et al*, 2008). The disease is frequently diagnosed at a late stage and despite advances in medical therapy, approximately 90% of patients die within a year of diagnosis (Stathis and Moore, 2010). The 5-year survival rate is only 3% (Rosewicz and Wiedenmann, 1997; American Cancer Society, 2007). Surgery is the only curative treatment option available, but is often precluded by local invasive growth and early metastatic spread to regional lymph nodes and distant organs (Jura *et al*, 2005; Mihaljevic *et al*, 2010). Up to now, sequential stages of disease initiation and progression have been characterised at the molecular level by activating mutations in oncogenes (e.g. *K-Ras*) and functional inactivation of tumour-suppressor genes (e.g. *P16*, *TP53* and *DPC4*) (Maitra and Hruban, 2008).

The *TP53* gene is the most frequently mutated gene identified in human cancer (Soussi and Lozano, 2005) and a 50–70% mutation rate was reported in pancreatic adenocarcinoma, ranking this disease among those with the highest incidence of *TP53* inactivation (Rozenblum *et al*, 1997). The functional importance of *TP53* inactivation for pancreatic cancer formation has been convincingly demonstrated in several genetically engineered mouse models of pancreatic cancer, where loss of *p53* cooperates with *p16* and *K-Ras* alterations (Hruban *et al*, 2006). *TP53* is mostly inactivated by a single mutation within the DNA-binding domain resulting in a functionally impaired full-length protein (Soussi and Lozano, 2005; Goh *et al*, 2011).

It is well known that TP53 is activated by cell stress, arresting the cell during the cell cycle, repairing damaged DNA or inducing apoptosis as the cell requires (Braithwaite and Prives, 2006; Riley *et al*, 2008). p21^WAF1/CIP1^ has been defined as a major effector, which is transcriptionally induced by wild-type (wt) TP53, but not by mutated TP53 (Vikhanskaya *et al*, 2007). p21^WAF1/CIP1^ interferes in the cell cycle at different time points by binding the complexes of cyclins and cyclin-dependent kinases (Coqueret, 2003). Besides this, TP53 is able to affect the vascularisation of tumours (Dameron *et al*, 1994; Semenza, 2001; Mukhopadhyay and Datta, 2004) by regulating the expression of angioregulatory factors (e.g. VEGF, Tsp-1 or Hif-1*α*) and their receptors in a direct as well as in an indirect manner. Analyses of precursor lesions of invasive ductal pancreatic cancer have established TP53 alterations in PanIN3 lesions, suggesting that they occur at late stages of the transformation process (Bardeesy and DePinho, 2002).

Restoration of the wt TP53 function has been achieved in different tumour cell systems, including pancreatic cancer cells (Lang *et al*, 1998; Cascallo *et al*, 1999; Ghaneh *et al*, 2001), and resulted in cell cycle arrest or apoptosis, and insensitivity to radiation (Liu *et al*, 2009) or chemotherapy (Sullivan *et al*, 2004). Wt TP53 reexpression similarly inhibited xenograft growth *in vivo* in mouse models (Ghaneh *et al*, 2001; Xue *et al*, 2007).

Although these approaches were suitable to address the role of TP53 for early aspects of tumour formation, the requirement of persistent TP53 inactivation in already established tumours has only recently been addressed and confirmed (Martins *et al*, 2006; Ventura *et al*, 2007; Xue *et al*, 2007). However, so far, data for the effects of wt TP53 reconstitution in established pancreatic tumours are not available.

Our study sets up a model for investigating the effects of wt TP53 restoration at different stages of pancreatic tumour growth using an orthotopic *in vivo* approach. A tetracycline-inducible wt TP53 was stably transfected into the pancreatic carcinoma cell lines DanG and MiaPaCa-2 bearing *TP53* mutations. Induction of wt TP53 reduced cell proliferation *in vitro* by induction of p21^WAF1/CIP1^ and potently inhibited growth of orthotopic xenografts *in vivo*. Despite the reduction of primary tumour growth, an increase in the number of lymphatic vessels and in lymph node metastasis occurred.

## Materials and methods

### Cell culture

MiaPaCa-2 cells were obtained from ATCC (Manassas, VA, USA) and DanG cells were obtained from DSMZ (Braunschweig, Germany). For transfected variants, frozen batches of stock generated shortly after receipt of cell lines from the repositories were used. For experiments on transfected cell lines, frozen stocks from early passages were kept in culture for a maximum of 4 months. MiaPaCa-2 cells were maintained in DMEM and DanG cells were maintained in RPMI medium. Media were supplemented with 10% foetal bovine serum. All cells were cultured at 37 °C with 5% CO_2_. To induce wt *TP53* expression, doxycycline (Dox) was added to the medium every 2 days to a final concentration of 1 *μ*g ml^−1^ (i.e., +Dox). The cells were treated additionally with 500 U ml^−1^ interferon-*γ* (IFN-*γ*, Roche Applied Science, Mannheim, Germany) as control during the apoptosis assay or with gemcitabine (Gemzar; Eli Lilly and Company, Indianapolis, IN, USA) in chemotherapy response assays.

### Plasmids and DNA transfection

The wt *TP53* cDNA was obtained from the plasmid pcDNA3.1±TP53 (kindly provided by DP Xirodimas, Department of Surgery and Molecular Oncology, Ninewells Hospital and Medical School, University of Dundee, Dundee, UK) (Xirodimas *et al*, 2001) using PCR. The sequences of the primers were: 5′-GCGGCCGCATGGAGGAGCCGCAGTCA-3′, sense and 5′-GCGGCCGCTCACTTATCGTCGTCATCCTTGTAATCGTCTGAGTCAGGCCC-3′, antisense. Both primers contained a Not I restriction site and the antisense primer additionally contained a *FLAG* sequence. The PCR mixture consisted of 0.5 *μ*l PFU-DNA-polymerase (Stratagene/Agilent, Waldbronn, Germany), 1 × PCR buffer (Mg^2+^ Plus), 0.2 mM of each dNTP, 0.2 mM of each primer and 10 ng vector DNA in a total volume of 25 *μ*l. Amplification parameters were as follows: 94 °C for 3 min, followed by two cycles, each of which comprised a melting step at 94 °C for 45 s, an annealing step at 48 °C for 45 s, extension at 72 °C for 2.5 min, followed by 28 cycles, each of which comprised a melting step at 94 °C for 45 s, an annealing step at 52 °C for 45 s, extension at 72 °C for 2.5 min, and finally at 72 °C for 10 min. PCR product was inserted into the TOPO TA cloning vector (version M) (Invitrogen, Berlin, Germany). The *TP53-FLAG* cDNA was restricted by endonuclease Not I (Roche), inserted in pcDNA4/TO and identified by restriction with endonuclease *Pvu*II (Roche). MiaPaCa-2 and DanG cells were serially transfected with the plasmids pcDNA6/TR and pcDNA/TO-TP53FLAG using the Effectene transfection kit (Qiagen, Hilden, Germany). Transfectants were selected in the presence of blasticidin (5 *μ*g ml^−1^) and zeocin (500 *μ*g ml^−1^) (Invitrogen). Individual clones of stable double transfectants, namely MiaPaCa-2-TREx-TP53 and DanG-TREx-TP53, were selected for further analyses (see Invitrogen User Manual).

### Determination of proliferation and cellular senescence

Log-phase MiaPaCa-2-TREx-TP53 and DanG-TREx-TP53 cells growing in –Dox medium were seeded at a density of 2 × 10^5^ cells per well in a 24-well plate. Cells were allowed to adhere (16 h) and then cultured in the presence or absence of Dox (1 *μ*g ml^−1^).

Cell proliferation was measured at 24, 48, 72 and 96 h by the alamarBlue assay, purchased from BioSource International Inc. (Camarillo, CA, USA). To determine the antiproliferative action of gemcitabine, viable cells were counted manually using a haemocytometer.

For detection of cellular senescence, cells were treated for 48 h −/+Dox, fixed and assayed for beta galactosidase activity as recommenced by the manufacturer (Sigma-Aldrich, St Louis, MO, USA).

### Flow cytometry

After ethanol fixation and propidium iodide staining as described (Detjen *et al*, 2001), 10 000 cells per assay and time point were analysed by a FACSCalibur Flow Cytometry System (Becton Dickinson, Heidelberg, Germany), using the CellQuest software (Becton Dickinson).

### Western blot analysis

Cell lysates were prepared and blotted as described (Plath *et al*, 2000). Proteins were transferred to polyvinylidene difluoride membrane (Millipore, Billerica, MA, USA), which after blocking (3 or 5% milk in phosphate- or tris-buffered saline with 0.1 or 0.05% Tween-20) were treated with the following primary antibodies: anti-FLAG M2 (1 : 500 dilution; Sigma-Aldrich), anti-p53 (1 : 1000 dilution; DO-1; Santa Cruz Biotechnology, Danvers, MA, USA), anti-p21 (1 : 200 dilution; Oncogen, La Jolla, CA, USA), anti-cyclin A (1 : 200 dilution; Upstate/Millipore), anti-cyclin-dependent kinase-2 (1 : 1000 dilution; Santa Cruz Biotechnology) and anti-*β* actin (1 : 1000 dilution, Sigma-Aldrich). Western blots were developed with the enhanced chemiluminescence reagent (oxidising and enhanced luminol; PerkinElmer Life Sciences, Boston, MA, USA).

### Cleavage of poly(ADP-ribose)polymerase

Aliquots of 4 × 10^5^ cells were lysed in 200 *μ*l buffer containing 62.5 mM Tris/HCL (pH 6.8), 6 M urea, 10% glycerol, 2% SDS, 0.00125% bromophenol blue and 5% *β*-mercaptoethanol. After sonification for 15 s and incubation at 65°C for 15 min, lysates were separated by SDS–PAGE. Poly(ADP-ribose)polymerase immunodetection was carried out as described above using the anti-poly(ADP-ribose)polymerase antibody (Oncogen) at a dilution of 1 : 500.

### Animals and tumour cell transplantation

The studies were performed in accordance with a protocol approved by the regional animal research committee. The *in vivo* procedures were in compliance with the UKCCCR guidelines. Female 6- to 8-week-old nude BALB/c mice were used and the orthotopic transplantation protocol was performed as described (Alves *et al*, 2001). To induce wt TP53 expression *in vivo*, the mice were treated with Dox (Invitrogen) in the drinking water to a final concentration of 1 mg ml^−1^ (i.e. +Dox), which was changed every second day and stored in a lightproof bottle. Autopsies were performed and all findings, such as invasion into the duodenum and stomach, number and location of macroscopic metastases and the weight of the primary tumour, were recorded. In addition, liver hilus and mesenterial lymph nodes were routinely collected for further examination. Directly after this, section tissues were frozen in liquid nitrogen.

### Immunohistochemistry

For immunohistochemistry, 7 *μ*m-thick cryosections were fixed in 4% paraformaldehyde/PBS, pH 7.0 for 20 min at room temperature. After treatment with 0.3% hydrogen peroxide for 10 min, the cryosections were incubated for 1 h at room temperature with anti-LYVE-1 (1 : 100; RELIA Tech, Wolfenbüttel, Germany), anti-CD31 (1 : 100; BD Biosciences, Franklin Lakes, NJ, USA), anti-CD34 (1 : 10; BD Biosciences) or anti-pan-cytokeratin-HRP (1 : 50; Santa Cruz Biotechnology) antibody followed by incubation with a secondary biotinylated antibody. Immunoperoxidase staining was performed with an avidin–biotin complex method (Vectastain Elite ABC kit; Vector Laboratories, Inc., Burlingame, CA, USA) and AEC substrate. For detection of the FLAG-tag, the fixed crysections were stained using the Animal Research kit (DakoCytomation, Glostrup, Denmark) and the anti-FLAG M2 antibody (Santa Cruz Biotechnology). All sections were counterstained with hematoxylin (Vector Laboratories, Inc.). Specificity controls were performed without primary antibody.

### Angiogenesis and lymphangiogenesis

For quantification of microvessel density (MVD) and lymphatic vessel density (LVD), the average numbers of CD31 or CD34 and LYVE-1 positive vessels from three areas (each 0.6 mm^2^) of maximal vascular density (vascular hotspots) were counted and the mean average was determined (Weidner *et al*, 1991).

### Statistical analysis

Data were analysed by *t*-test and Fisher's exact test using GraphPad Prism statistical software (GraphPad Software Inc., San Diego, CA, USA). Differences were considered significant at *P*<0.05.

## Results

### Inducible wt TP53 expression in pancreatic carcinoma cell lines led to growth inhibition by G1 cell-cycle arrest

**Figure 1:**
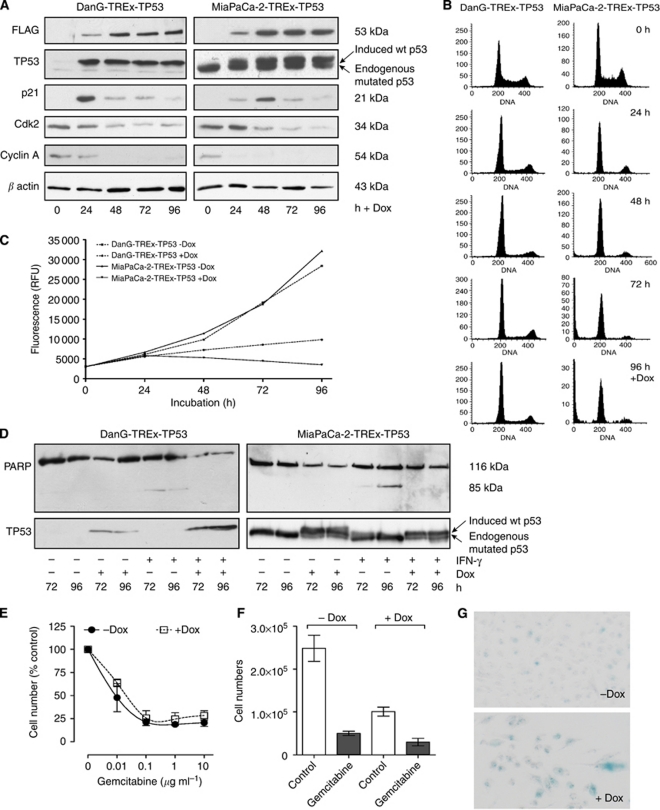
Generation of DanG-TREx-TP53 and MiaPaCa-2-TREx-TP53 cells with Dox-inducible expression of functionally active wt TP53. (**A**) The plasmids pcDNA6/TR and pcDNA4/TO-TP53FLAG were sequentially transfected into DanG and MiaPaCa-2 cells bearing mutant endogenous p53 and clones with inducible expression of wt TP53 were selected (DanG-TREx-TP53 and MiaPaCa-2-TREx-TP53). Cells were cultured for 96 h in the presence or absence of Dox and lysates were prepared for western blot analysis of FLAG (upper panel) and TP53 (second panel) at the indicated time points. Equal amounts of protein (10 *μ*g) were separated on SDS polyacrylamide gel electrophoresis (PAGE). Furthermore, effects of Dox-induced wt TP53 expression on downstream targets were investigated by western blot analysis: p21^WAF1/CIP1^, cyclin-dependent kinase-2, cyclin A and *β*-actin (lower panels). Equal amounts of protein (30 *μ*g) were separated on SDS–PAGE. (**B**) Effects on cell cycle after wt TP53 expression in DanG-TREx-TP53 and MiaPaCa-2-TREx-TP53 were analysed by FACS over a time course of 96 h. At every 24 h, cells were fixed with 70% ethanol and stained with propidium iodide. (**C**) Time-dependent proliferation of DanG-TREx-TP53 and MiaPaCa-2-TREx-TP53 cells in the presence or absence of Dox as determined by alamar blue assay. At every 24 h, alamar blue dye was applied to the media of cells and fluorescence was determined as an indirect measurement of cell numbers. (**D**) Immunoblot demonstrating expression of poly(ADP-ribose)polymerase (p116) and/or its apoptosis-related cleavage product p85 in untreated controls and cells that had been treated with Dox, IFN-*γ* or a combination of both for 72 or 96 h. Whole-cell lysates were separated by 7.5% SDS–PAGE. (**E**) The diagram depicts the concentration dependence of the growth inhibition by gemcitabine with data presented as a percentage of the respective controls (vehicle and +Dox, respectively). (**F**) The response of the MiaPaCa-2-TREx-TP53 cells to gemcitabine was evaluated by determination of cell numbers. The cells were treated with −/+Dox and −/+gemcitabine, as indicated, for 48 h. The diagram presents cell numbers after treatment with or without 10 *μ*g ml^−1^ gemcitabine. (**G**) Cellular senescence (*β*-Gal activity) in MiaPaCa-2-TREx-TP53 cells was detected by a cell senescence assay after −/+Dox treatment. Senescence-positive cells appeared blue ( × 200 magnification). A representative experiment out of two is shown.

The pancreatic cancer cell lines DanG and MiaPaCa-2 harbour mutated *TP53* alleles (Moore *et al*, 2001), resulting in the accumulation of a stabilised mutant protein (Midgley and Lane, 1997), which is easily detected by western blot analysis with a TP53 antibody (Figure 1A in the lane without Dox). In order to inducibly restore wt TP53 in these pancreatic cancer cell lines, the tetracycline inducible T-REx system from Invitrogen was used. The cDNA of wt *TP53* was cloned into the tet-responsive vector pcDNA4/TO, tagged with a FLAG-tag at the C-terminus for better detection, and sequenced to confirm correct wt *TP53-FLAG* insertion as well as the presence of the polymorphism proline in codon 72. After successful transfection, two clones with strong induction of wt TP53 and very low background were selected based on immunodetection of the FLAG-tag in western blot analyses (DanG-TREx-TP53 and MiaPaCa-2-TREx-TP53) (Figure 1A, first panel). Upon Dox treatment, these clones revealed a prominent and persistent expression of wt TP53 over a 4-day period (Figure 1A). As expected, the p53 antibody also detected the endogenously expressed mutated TP53 protein, which corresponds to the lower, faster migrating band (Figure 1A, second panel). To investigate the functional outcome of wt TP53 induction, proliferation was measured. Upon wt TP53 expression, DanG-TREx-TP53 and the MiaPaCa-2-TREx-TP53 cells showed a distinct growth inhibition (Figure 1C), suggesting that exogenously induced wt TP53 was functionally intact in these pancreatic carcinoma cell lines.

To further characterise TP53 function in the transfected cells, we conducted cell cycle analysis using FACS. Within 24 h of wt TP53 induction, cells accumulated in the G1 phase and decreased in the S and G2 phases. This redistribution was maintained throughout the 96-h time period of wt TP53 reexpression (Figure 1B), suggesting that expression of wt TP53 resulted in G1 cell cycle arrest. Consistent with the cell cycle redistribution, we furthermore observed a prominent induction of the endogenous cyclin-dependent kinase inhibitor p21^WAF1/CIP1^ in Dox-treated cells, which was followed by a reduction of cyclin A and cyclin-dependent kinase-2 protein levels (Figure 1A).

Although the pre-G1 fractions from Dox-treated cells were comparable to their respective controls, TP53 induction *per se* did not appear to be associated with apoptosis induction in the pancreatic cancer cell lines utilised. In addition, we aimed to determine whether wt TP53-dependent growth arrest might confer apoptosis protection towards a known pro-apoptotic stimulus (Detjen *et al*, 2001). Therefore, DanG-TREx-TP53 and MiaPaCa-2-TREx-TP53 cells were treated either with Dox, IFN-*γ* or a combination of both, and the 85 kDa poly(ADP-ribose)polymerase cleavage product that is indicative of apoptosis was identified by western blot analysis. After 72 and 96 h, the cleavage product at 85 kDa was observed exclusively in cells treated with IFN-*γ*, but not in cells that had been induced to express TP53 (Figure 1D). Thus, the IFN-*γ*-induced apoptosis reaction was inhibited in cells with reexpressed wt TP53.

Activation of wt TP53 was previously shown to enhance survival of tumour cells treated with nucleoside analogues, such as gemcitabine. Therefore, we determined the effects of gemcitabine in MiaPaCa-2-TREx-TP53 cells in the presence or absence of Dox. When induction of TP53 was started at the time of gemcitabine application, maximal growth inhibition due to the nucleoside analogue relative to the respective controls was comparable and the dose-response profile was not changed by TP53 (Figure 1E). We also tested the effect of gemcitabine in cells that were preincubated with Dox for 48 h, such that TP53 was induced prior to gemcitabine treatment. Although the profound growth inhibition due to TP53 was further increased by gemcitabine, induction of the tumour suppressor did not appreciably protect the cells from gemcitabine or sensitise them to treatment (data not shown or Figure 1F).

The profound and persistent growth inhibition without induction of apoptosis suggested permanent cell cycle withdrawal as occurs upon induction of cellular senescence. Indeed, Dox-treated MiaPaCa-2-TREx-TP53 presented increased SA-*β*-Gal activity, when compared with control cells without TP53 induction, confirming a senescence phenotype (Figure 1G).

### Pancreatic tumour growth, macroscopic metastasis and invasion were reduced in mice with wt TP53 reconstitution

As our *in vitro* characterisation had confirmed that we were able to efficiently control the expression of a functional wt TP53, we next aimed to determine the consequences of wt TP53 induction *in vivo* in an orthotopic context using the MiaPaCa-2-TREx-TP53 cell system. The MiaPaCa-2 system was chosen because it offers a better opportunity to evaluate invasive and metastatic spread of the primary tumours when compared with DanG tumours, which are characterised by rapid disease progression due to tumour cachexia. Previous studies had indicated that MiaPaCa-2-TREx cells readily formed tumours, which were not affected by Dox treatment (Schulz *et al*, 2008).

**Figure 2:**
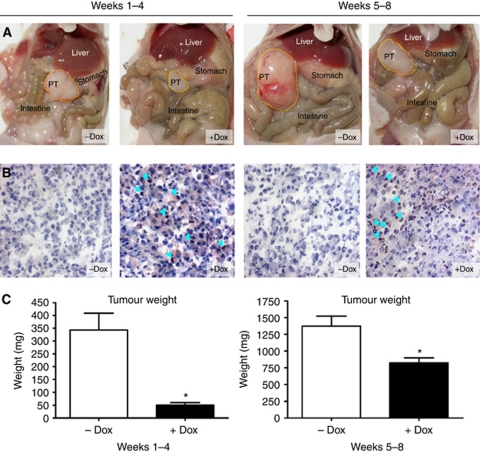
Induction of wt TP53 expression reduced primary tumour growth in orthotopic MiaPaCa-2 pancreatic carcinomas at both time points. MiaPaCa-2-TREx-TP53 cells were grown orthotopically in the pancreas of mice treated with or without Dox as indicated. At the end of treatment (either 4 weeks or 8 weeks) tumour weights were determined. (**A**) Open situs of representative mice from each control (−Dox) and treatment group (+Dox). (**B**) Conformation of wt TP53 induction *in vivo*. Primary tumours were analysed for TP53 expression by immunohistochemistry using an antibody against the FLAG-tag. Examples of wt TP53 expression at 4 and 8 weeks of tumour growth, with detection of the wt TP53-FLAG protein in the nucleus of tumour cells from Dox-treated mice indicated by the light blue arrows. In contrast, no signal was obtained in control tumours (−Dox) (images at × 200 magnification). (**C**) Summary of primary tumour weights of Dox-treated or -untreated animals receiving Dox either in weeks 1–4 or in weeks 5–8. Data represent mean±s.e.m. for each group. ^*^P<0.05.

We then proceeded to induce wt TP53 expression in tumour-bearing mice during different periods of tumour growth, thereby investigating (i) the function of wt TP53 in the initial steps of tumour formation and early tumour growth and (ii) the function of wt TP53 for the maintenance of already established tumours, which more closely reflects a potential therapeutic situation. Accordingly, wt TP53 was induced throughout 4 weeks from the time of implantation or from week 5 through week 8 (Table 1). After 4 or 8 weeks, the mice were killed and primary tumours were removed to determine the weight. Representative pictures of the opened abdominal situs in mice from different groups are shown in Figure 2A. The mean weights of primary tumours were significantly lower in Dox-treated animals at both timepoints of wt TP53 induction. Mice with early induction (weeks 1–4) revealed an 85% reduction of the primary tumour weight compared with their control animals. In addition, we were unable to detect primary tumours in 3 out of 12 animals from the treatment group during autopsy (Figure 2C). We hypothesise that this inhibition of tumour growth was due to the reexpression of wt TP53. Accordingly, we confirmed wt TP53 expression in cryosections of primary tumours of Dox-treated animals by immunohistochemistry with an antibody against the FLAG-tag (Figure 2B). In total, successful *in vivo* induction was evident in all nine tumour-bearing mice with MiaPaCa-2-TREx-TP53 orthotopic tumours and absent in all control animals.

Mice with late Dox treatment (weeks 5–8) similarly presented a significant reduction of primary tumour weight compared with their control animals (Figure 2C), extending the antitumour activity of wt TP53 to already established pancreatic tumours. Successful wt TP53 induction in Dox-treated mice was again confirmed by immunohistochemistry, as described above (Figure 2B). Wt TP53 was absent in 8 out of 12 mice in the control group. However, a weak immunoreactivity was observed in the remaining four control tumours, possibly due to promoter leakage.

Of the tumours with confirmed wt TP53 expression only three invaded into surrounding tissues, such as stomach and duodenum (Table 1), whereas at least six tumours from the control group presented with invasive growth. In addition, several control mice – none with TP53 reactivity – presented macroscopic metastasis in liver, lung, gut, stomach, kidney and spleen (Table 1). These distant metastases were detected almost exclusively after 8 weeks of tumour growth.

In summary, reexpression of wt TP53 in pancreatic cancer cells significantly inhibited orthotopic tumour growth *in vivo* in a similar way to the growth inhibitory effects *in vitro*.

### Induction of wt TP53 expression increased vascularisation in the early stages of tumour growth

**Figure 3:**
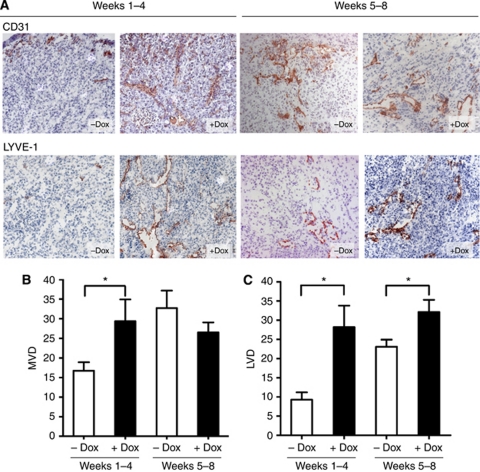
Effects of wt TP53 restitution on vascularisation in orthotopic MiaPaCa-2-TREx-TP53 pancreatic carcinomas. Cryosection of the tumours from control (−Dox) and treatment groups (+Dox) were stained with endothelial marker CD31 orlymphatic endothelial marker LYVE-1. To determine vessel densities, the number of CD31- and LYVE-1-expressing vessels were quantitated from hotspot areas, as described in Materials and Methods. (**A**) Shown are representative CD31 (upper panel) and LYVE-1 stainings (lower panel) from −Dox- and +Dox-treated tumours of both groups (**B** and **C**). Summary of the quantitative evaluation of the results is given as MVD or LVD, ^*^P<0.05 (images at × 100 magnification).

Induction of wt TP53 expression significantly inhibited MiaPaCa-2-TREx-TP53 tumour growth, which may have resulted from direct antiproliferative or from indirect mechanisms. As vascularisation is a prerequisite for tumour growth, we examined whether wt TP53 influenced angiogenesis. Cryosections of the primary tumours were stained with CD31 to detect endothelial cells and average MVD was determined for each group (Figure 3). After 4 weeks of tumour growth, the mean MVD in the control animals averaged 17 vessels, indicating that substantial neo-vascularisation had occurred. Unexpectedly, the tumours expressing wt TP53 presented higher MVD values (29 *vs* 17, *P*=0.0196, Figure 3B). By the end of 8 weeks, mean MVD of control mice had increased to 33 vessels and no longer differed from the MVD determined in tumours with wt TP53 induction (33 *vs* 27, *P*=0.2298, Figure 3B). Importantly, tumour size *per se* did not correlate with MVD (data not shown), indicating that changes in MVD values in the Dox-treated mice groups most likely reflected a specific biological effect of wt TP53 reconstitution.

Haemangiogenesis was then more specifically determined by immunohistochemistry with a CD34 antibody detecting a glycoprotein on the surface of vascular endothelial cells in MiaPaCa-2-TREx-TP53 tumours that were grown for 8 weeks. MVD averaged 65 vessels in control tumours *vs* 48 in Dox-treated tumours (*P*=0.154), thus reflecting the results obtained by CD31 staining.

### Induction of wt TP53 increases lymphangiogenesis in pancreatic tumours

MVD determined by CD31 staining represents endothelium from both blood vessels and lymph vessels. In order to distinguish between haemangiogenesis and lymphangiogenesis, the latter was determined separately based on immunostaining with LYVE-1, an antibody detecting the hyaluronan receptor-1 of lymphatic vessels (Figure 3). Strikingly, all tumours with wt TP53 reconstitution contained more LYVE-1-positive lymphatic vessels than control tumours, increasing the LVD by 203% at 4 weeks (28 *vs* 9, *P*=0.0011) and by 39% at 8 weeks (32 *vs* 23, *P*=0.0214) (Figure 3D). Similar to MVD, LVD did not *per se* correlate with tumour size (data not shown).

**Figure 4:**
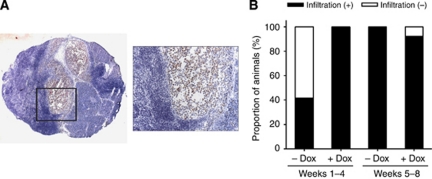
(**A**) Effects of wt TP53 induction on metastatic spread of MiaPaCa-2 cells to the liver hilus lymph node. Micro-metastatic spread of MiaPaCa-2-TREx-TP53 cells was evaluated based on detection of human cytokeratin in available mouse lymph nodes (*n*=7). Inspection of lymph node metastasis, which was stained by pan-cytokeratin antibody. Representative picture of a lymph node stained with an anti-human pan-cytokeratin antibody, revealing positive human tumour cells (stained red) surrounded by lymphocytes (image at × 25 magnification and × 200 magnification (image inset)). (**B**) Quantitative analysis of lymph node metastasis. Data represent the proportion of animals with presence of lymph node metastasis in the liver hilus lymph node, compared between the treatment (+Dox) and control group (−Dox) of different time periods with wt TP53 reexpression.

As lymphangiogenesis has emerged as a key determinant of lymphatic metastasis, we proceeded to determine the effect of wt TP53 reexpression on lymph node metastasis in MiaPaCa-2-TREx-TP53 tumours. For this purpose, the lymph nodes from the liver hilus were stained by an anti-human pan-cytokeratin antibody (Figure 4A). At 4 weeks of tumour growth, all available liver hilus lymph nodes of mice from the Dox-treated group contained human epithelial cells, indicative of lymph node metastasis formation. In contrast, only 5 out of 12 lymph nodes of control animals revealed human cells, suggesting an increased incidence of lymph node invasion in mice with wt TP53 reexpression at this stage of tumour progression. At 8 weeks, almost all liver hilus lymph nodes, even in the control mice, showed human cell infiltration (Figure 4B). Mesenterial lymph nodes were also evaluated, but had not been invaded by metastatic cells in any of the mice (data not shown). In summary, wt TP53 restitution had increased lymphangiogenesis and promoted early lymph node invasion.

## Discussion

Inactivation of wt TP53 function is a common event during tumour progression, occurring at an above-average rate in pancreatic adenocarcinomas. Restoration of this famous ‘gatekeeper’ has been a long-standing goal in cancer biology, and promising strategies and drugs have moved this concept forward towards clinical trials (Vazquez *et al*, 2008). Here, we mainly addressed the consequences of wt TP53 reexpression in pancreatic cancer cells *in vivo* in an orthotopic tissue context. Consistent with previous work in subcutaneous xenograft models of pancreatic carcinomas (Ghaneh *et al*, 2001; Cascallo *et al*, 2005), we found that wt TP53 restoration distinctly reduces primary tumour growth during both the early and late stages of orthotopic tumour growth. Thus, continued inactivation of the tumour suppressor was required for the maintenance of malignant tumour growth in orthotopic MiaPaCa-2 tumours. This is in line with earlier results from experimental approaches using genetically engineered mice to elicit wt p53 expression in oncogene-driven lymphomas and sarcomas (Martins *et al*, 2006; Ventura *et al*, 2007), and in liver tumours with inducible p53 expression in the tumour cells (Xue *et al*, 2007). These studies furthermore delineated massive apoptosis induction or cell cycle withdrawal as alternative outcomes of p53, depending on the tumour type examined. Although wt TP53 has previously been reintroduced in permanent pancreatic cancer cell lines (Lang *et al*, 1998; Ghaneh *et al*, 2001), little is known about the consequences of wt TP53 reexpression for more complex, context-driven aspects of pancreatic cancer growth, upon which we focused in the current study. For this purpose, we combined a tetracycline-inducible expression system with an orthotopic approach, which allowed several potential obstacles to be overcome: the inducible system permitted selection, expansion and continuous culture of stable clones, while avoiding the selective pressure expected from overexpression of a tumour suppressor and mediator of cellular senescence. Upon addition of Dox, functionally relevant expression of wt TP53 in DanG-TREx-TP53 and MiaPaCa-2-TREx-TP53 was reproducibly obtained, as judged from the ability to inhibit G1 cell cycle progression by upregulation of p21^WAF1/CIP1^ (Vikhanskaya *et al*, 2007). Although the proapoptotic function of TP53 is well established, we failed to detect a major increase in apoptosis following wt TP53. Previous studies have found an association between the proline/arginine polymorphism in codon 72 and the induction of cell cycle inhibition or apoptosis. As the current study utilised a construct carrying the proline variant, our observation of growth arrest in the pancreatic cancer cell lines is in excellent agreement with the proposed differential biological effects (Sullivan *et al*, 2004). Moreover, cells with restitution of TP53 were not sensitised to apoptosis induction by IFN-*γ* or to the cytotoxic effects of gemcitabine. This phenotype fits well with our observation of cellular senescence in MiaPaCa-2 cells upon induction of TP53. Senescence may also represent one mechanism responsible for inhibition of MiaPaCa-2 tumour growth *in vivo*. For instance, either senescence or apoptosis were responsible for tumour regression, when TP53 was temporarily reexpressed in autochthonous tumours in mice using a Cre-*loxP*-based strategy (Ventura *et al*, 2007). Thus, both the tissue-specific tumour context and the type of mutation impinge on the outcome of TP53 activation.

For several reasons, the orthotopic approach pursued in the current study seemed of particular benefit. First, primary tumours were allowed to form and progress within their correct microenvironment. Thus, tissue-specific interactions between tumour cells and the stromal and vascular compartments, which are increasingly recognised as major determinants of the tumour phenotype, were maintained (Blouw *et al*, 2003; Chu *et al*, 2007). Second, orthotopic pancreatic tumours metastasise via clinically relevant routes and tumour spread causes complications also seen in human patients with pancreatic cancer, thereby offering information that is unavailable from subcutaneous xenografts (Li *et al*, 2009). Third, the orthotopic approach in nude mice allowed human carcinoma cells to be utilised, which are expected to reflect the characteristic molecular makeup of the human disease. Thus, the orthotopic model offers the potential to obtain data on the effects of wt TP53 restitution in pancreatic cancer with improved clinical relevance when compared with subcutaneous xenografts (Killion *et al*, 1998).

The combination of the inducible expression system with an orthotopic approach was feasible, as shown by successful detection of wt TP53 in tumours of Dox-treated mice. However, at 8 weeks after transplantation, almost one-third of the control animals revealed wt TP53 immunoreactivity, suggesting that some promoter leakage occurred even in the absence of Dox. Thus, though *in vivo* control of wt TP53 can be achieved, monitoring of target expression is mandatory.

The MiaPaCa-2-TREx-TP53 orthotopic primary tumours were well vascularised, as has been reported for clinical specimens of PDAC (von Marschall *et al*, 2000). Furthermore, the MVD recorded in the present study matched the results obtained in previous studies using orthotopic MiaPaCa-2 xenografts (Schulz *et al*, 2008). Thus, it most likely reflected a characteristic feature of MiaPaCa-2 pancreatic tumours rather than a random feature of a xenograft tumour grown from cells that had undergone multiple transfections. A substantial part of the tumour vasculature could be further specified as lymphendothelial, providing us with the opportunity to also determine lymphangiogenesis, which has become a focus of research because of the proposed role in lymphatic metastasis (Stacker *et al*, 2002; Von Marschall *et al*, 2005).

Induction of wt TP53 *in vivo* profoundly affected the angiogenic phenotype of MiaPaCa-2-TREx-TP53 tumours. Interestingly, the LVDs obtained from tumours with early induction of wt TP53 pointed to a significantly increased lymphangiogenesis. This unexpected observation fits well with the high incidence of lymphatic metastases in tumours with wt TP53 induction during the initial 4 weeks of tumour growth. The high incidence of lymph node metastasis in tumours with TP53 induction is remarkable, as they were derived from tumours with <100 mg tumour mass, which in our experience with the MiaPaCa-2 tumour cell model do not yet metastasise. An increase in LVD was also detected in tumours with late wt TP53 reexpression. By that time, mice of both groups had almost uniformly developed lymph node metastasis, suggesting that TP53 was unable to suppress lymphatic metastasis, despite the prominent reduction of growth in the primary tumours.

A link between TP53 and regulation of angiogenesis has been previously reported (Teodoro *et al*, 2007). Mutation of *TP53* was found to be associated with high levels of VEGF expression and/or high MVD in clinical specimens from various cancer entities including colorectal, breast and prostate cancer. In an experimental setting, stabilised wt TP53 was shown to bind to HIF-1*α*, resulting in accelerated degradation of HIF-1*α*, and thus in a restriction of hypoxia-driven tumour neoangiogenesis. Furthermore, TP53 was reported to act as a transcriptional repressor of proangiogenic growth factors and activator of angiogenesis inhibitors (Teodoro *et al*, 2007). These concepts are difficult to reconcile with our observation of an increase in (lymph)angiogenesis following the *in vivo* induction of TP53. We were also unable to detect an inhibition of VEGF production in MiaPaCa-2 cells following reexpression of wt TP53 (data not shown). However, a possible explanation for the apparent discrepancy may come from a study that used RNA interference to conditionally regulate endogenous *TP53* expression in a mosaic mouse model of liver cancer. TP53 induction not only impaired tumour growth, as was anticipated, but also elicited tumour regression. The anti-tumour effect was associated with senescence induction and a pronounced inflammatory response of the innate immune system (Xue *et al*, 2007). At the level of tumour vascularisation, a perivascular infiltration developed into vasculitis and ultimately resulted in the destruction of the tumour vasculature. Thus, wt TP53 reconstitution was found to be associated with an activation of the tumour vasculature. It is currently unclear whether the inflammatory response is a general response following reactivation of wt TP53 or restricted to distinct tumour entities. Given that chronic inflammation has been shown to stimulate lymphangiogenesis in the inflamed tissue as well as in the draining lymph node (Halin *et al*, 2007), it is tempting to speculate that our observation may be a reflection of inflammatory lymphangiogenesis, a situation that was proposed to facilitate lymph node metastasis (Xue *et al*, 2007).

In summary, our study suggests that the beneficial effects of the antitumour activity of wt TP53 restoration may be accompanied by unexpected ‘side effects’. A careful delineation of participating mechanisms is of critical importance in view of the potential therapeutic use of TP53 mimetics.

## Figures and Tables

**Table 1:** Summary of the mouse treatment groups (number and time course) and results of tumour growth (weight, metastasis, invasion and vascularisation)

	**Group I (early)**		**Group II (late)**	
Supplements in the drinking water	No Dox	+Dox	No Dox	+Dox
Number of animals	13	12	13	13
Start of treatment	Directly after transplantation		Week 5	
Period of treatment	Weeks 1–4		Weeks 5–8	
Autopsy after	4 weeks		8 weeks	
Average tumour weight in mg	343±65.7	50.4±9.24	1374±149.3	822.2±76.6
Number of animals with macroscopic metastasis	1	0	4	0
Number of animals with invasion into duodenum and/or stomach	0	0	6	3

The deviation is given as s.e.m.

## References

[bib1] Alves F, Contag S, Missbach M, Kaspareit J, Nebendahl K, Borchers U, Heidrich B, Streich R, Hiddemann W (2001) An orthotopic model of ductal adenocarcinoma of the pancreas in severe combined immunodeficient mice representing all steps of the metastatic cascade. Pancreas 23: 227–235 11590317 10.1097/00006676-200110000-00002

[bib2] American Cancer Society (2007) Cancer Facts & Figures 2007. American Cancer Society: Atlanta, GA, pp 1–52

[bib3] Bardeesy N, DePinho RA (2002) Pancreatic cancer biology and genetics. Nat Rev Cancer 2: 897–909 12459728 10.1038/nrc949

[bib4] Blouw B, Song H, Tihan T, Bosze J, Ferrara N, Gerber HP, Johnson RS, Bergers G (2003) The hypoxic response of tumors is dependent on their microenvironment. Cancer Cell 4: 133–146 12957288 10.1016/s1535-6108(03)00194-6

[bib5] Braithwaite AW, Prives CL (2006) p53: more research and more questions. Cell Death Differ 13: 877–880 16708075 10.1038/sj.cdd.4401938

[bib6] Cascallo M, Calbo J, Capella G, Fillat C, Pastor-Anglada M, Mazo A (2005) Enhancement of gemcitabine-induced apoptosis by restoration of p53 function in human pancreatic tumors. Oncology 68: 179–189 16006755 10.1159/000086772

[bib7] Cascallo M, Mercade E, Capella G, Lluis F, Fillat C, Gomez-Foix AM, Mazo A (1999) Genetic background determines the response to adenovirus-mediated wild-type p53 expression in pancreatic tumor cells. Cancer Gene Ther 6: 428–436 10505853 10.1038/sj.cgt.7700070

[bib8] Chu GC, Kimmelman AC, Hezel AF, DePinho RA (2007) Stromal biology of pancreatic cancer. J Cell Biochem 101: 887–907 17266048 10.1002/jcb.21209

[bib9] Coqueret O (2003) New roles for p21 and p27 cell-cycle inhibitors: a function for each cell compartment? Trends Cell Biol 13: 65–70 12559756 10.1016/s0962-8924(02)00043-0

[bib10] Dameron KM, Volpert OV, Tainsky MA, Bouck N (1994) The p53 tumor suppressor gene inhibits angiogenesis by stimulating the production of thrombospondin. Cold Spring Harb Symp Quant Biol 59: 483–489 7587102 10.1101/sqb.1994.059.01.053

[bib11] Detjen KM, Farwig K, Welzel M, Wiedenmann B, Rosewicz S (2001) Interferon gamma inhibits growth of human pancreatic carcinoma cells via caspase-1 dependent induction of apoptosis. Gut 49: 251–262 11454803 10.1136/gut.49.2.251 PMC1728385

[bib12] Ghaneh P, Greenhalf W, Humphreys M, Wilson D, Zumstein L, Lemoine NR, Neoptolemos JP (2001) Adenovirus-mediated transfer of p53 and p16(INK4a) results in pancreatic cancer regression *in vitro* and *in vivo*. Gene Ther 8: 199–208 11313791 10.1038/sj.gt.3301394

[bib13] Goh AM, Coffill CR, Lane DP (2011) The role of mutant p53 in human cancer. J Pathol 223: 116–126 21125670 10.1002/path.2784

[bib14] Halin C, Tobler NE, Vigl B, Brown LF, Detmar M (2007) VEGF-A produced by chronically inflamed tissue induces lymphangiogenesis in draining lymph nodes. Blood 110: 3158–3167 17625067 10.1182/blood-2007-01-066811 PMC2200913

[bib15] Hruban RH, Adsay NV, Albores-Saavedra J, Anver MR, Biankin AV, Boivin GP, Furth EE, Furukawa T, Klein A, Klimstra DS, Kloppel G, Lauwers GY, Longnecker DS, Luttges J, Maitra A, Offerhaus GJ, Perez-Gallego L, Redston M, Tuveson DA (2006) Pathology of genetically engineered mouse models of pancreatic exocrine cancer: consensus report and recommendations. Cancer Res 66: 95–106 16397221 10.1158/0008-5472.CAN-05-2168

[bib16] Jemal A, Siegel R, Ward E, Hao Y, Xu J, Murray T, Thun MJ (2008) Cancer statistics, 2008. CA Cancer J Clin 58: 71–96 18287387 10.3322/CA.2007.0010

[bib17] Jura N, Archer H, Bar-Sagi D (2005) Chronic pancreatitis, pancreatic adenocarcinoma and the black box in-between. Cell Res 15: 72–77 15686632 10.1038/sj.cr.7290269

[bib18] Killion JJ, Radinsky R, Fidler IJ (1998) Orthotopic models are necessary to predict therapy of transplantable tumors in mice. Cancer Metastasis Rev 17: 279–284 10352881 10.1023/a:1006140513233

[bib19] Lang D, Miknyoczki SJ, Huang L, Ruggeri BA (1998) Stable reintroduction of wild-type p53 (MTmp53ts) causes the induction of apoptosis and neuroendocrine-like differentiation in human ductal pancreatic carcinoma cells. Oncogene 16: 1593–1602 9569027 10.1038/sj.onc.1201665

[bib20] Li M, Zhang Y, Bharadwaj U, Zhai QJ, Ahern CH, Fisher WE, Brunicardi FC, Logsdon CD, Chen C, Yao Q (2009) Down-regulation of ZIP4 by RNA interference inhibits pancreatic cancer growth and increases the survival of nude mice with pancreatic cancer xenografts. Clin Cancer Res 15: 5993–6001 19755388 10.1158/1078-0432.CCR-09-0557 PMC2756333

[bib21] Liu B, Zhang H, Duan X, Hao J, Xie Y, Zhou Q, Wang Y, Tian Y, Wang T (2009) Adenovirus-mediated wild-type p53 transfer radiosensitizes H1299 cells to subclinical-dose carbon-ion irradiation through the restoration of p53 function. Cancer Biother Radiopharm 24: 57–66 19243248 10.1089/cbr.2008.0514

[bib22] Maitra A, Hruban RH (2008) Pancreatic cancer. Annu Rev Pathol 3: 157–188 18039136 10.1146/annurev.pathmechdis.3.121806.154305 PMC2666336

[bib23] Martins CP, Brown-Swigart L, Evan GI (2006) Modeling the therapeutic efficacy of p53 restoration in tumors. Cell 127: 1323–1334 17182091 10.1016/j.cell.2006.12.007

[bib24] Midgley CA, Lane DP (1997) p53 protein stability in tumour cells is not determined by mutation but is dependent on Mdm2 binding. Oncogene 15: 1179–1189 9294611 10.1038/sj.onc.1201459

[bib25] Mihaljevic AL, Michalski CW, Friess H, Kleeff J (2010) Molecular mechanism of pancreatic cancer – understanding proliferation, invasion, and metastasis. Langenbecks Arch Surg 395: 295–308 20237938 10.1007/s00423-010-0622-5

[bib26] Moore PS, Sipos B, Orlandini S, Sorio C, Real FX, Lemoine NR, Gress T, Bassi C, Kloppel G, Kalthoff H, Ungefroren H, Lohr M, Scarpa A (2001) Genetic profile of 22 pancreatic carcinoma cell lines. Analysis of K-ras, p53, p16 and DPC4/Smad4. Virchows Arch 439: 798–802 11787853 10.1007/s004280100474

[bib27] Mukhopadhyay D, Datta K (2004) Multiple regulatory pathways of vascular permeability factor/vascular endothelial growth factor (VPF/VEGF) expression in tumors. Semin Cancer Biol 14: 123–130 15018896 10.1016/j.semcancer.2003.09.019

[bib28] Plath T, Detjen K, Welzel M, Von Marschall Z, Murphy D, Schirner M, Wiedenmann B, Rosewicz S (2000) A novel function for the tumor suppressor p16(INK4a): induction of anoikis via upregulation of the alpha(5)beta(1) fibronectin receptor. J Cell Biol 150: 1467–1478 10995450 10.1083/jcb.150.6.1467 PMC2150704

[bib29] Riley T, Sontag E, Chen P, Levine A (2008) Transcriptional control of human p53-regulated genes. Nat Rev Mol Cell Biol 9: 402–412 18431400 10.1038/nrm2395

[bib30] Rosewicz S, Wiedenmann B (1997) Pancreatic carcinoma. Lancet 349: 485–489 9040589 10.1016/s0140-6736(96)05523-7

[bib31] Rozenblum E, Schutte M, Goggins M, Hahn SA, Panzer S, Zahurak M, Goodman SN, Sohn TA, Hruban RH, Yeo CJ, Kern SE (1997) Tumor-suppressive pathways in pancreatic carcinoma. Cancer Res 57: 1731–1734 9135016

[bib32] Schulz P, Scholz A, Rexin A, Hauff P, Schirner M, Wiedenmann B, Detjen K (2008) Inducible re-expression of p16 in an orthotopic mouse model of pancreatic cancer inhibits lymphangiogenesis and lymphatic metastasis. Br J Cancer 99: 110–117 18577984 10.1038/sj.bjc.6604457 PMC2453030

[bib33] Semenza GL (2001) HIF-1 and mechanisms of hypoxia sensing. Curr Opin Cell Biol 13: 167–171 11248550 10.1016/s0955-0674(00)00194-0

[bib34] Soussi T, Lozano G (2005) p53 mutation heterogeneity in cancer. Biochem Biophys Res Commun 331: 834–842 15865939 10.1016/j.bbrc.2005.03.190

[bib35] Stacker SA, Achen MG, Jussila L, Baldwin ME, Alitalo K (2002) Lymphangiogenesis and cancer metastasis. Nat Rev Cancer 2: 573–583 12154350 10.1038/nrc863

[bib36] Stathis A, Moore MJ (2010) Advanced pancreatic carcinoma: current treatment and future challenges. Nat Rev Clin Oncol 7: 163–172 20101258 10.1038/nrclinonc.2009.236

[bib37] Sullivan A, Syed N, Gasco M, Bergamaschi D, Trigiante G, Attard M, Hiller L, Farrell PJ, Smith P, Lu X, Crook T (2004) Polymorphism in wild-type p53 modulates response to chemotherapy *in vitro* and *in vivo*. Oncogene 23: 3328–3337 15077186 10.1038/sj.onc.1207428

[bib38] Teodoro JG, Evans SK, Green MR (2007) Inhibition of tumor angiogenesis by p53: a new role for the guardian of the genome. J Mol Med 85: 1175–1186 17589818 10.1007/s00109-007-0221-2

[bib39] Vazquez A, Bond EE, Levine AJ, Bond GL (2008) The genetics of the p53 pathway, apoptosis and cancer therapy. Nat Rev Drug Discov 7: 979–987 19043449 10.1038/nrd2656

[bib40] Ventura A, Kirsch DG, McLaughlin ME, Tuveson DA, Grimm J, Lintault L, Newman J, Reczek EE, Weissleder R, Jacks T (2007) Restoration of p53 function leads to tumour regression *in vivo*. Nature 445: 661–665 17251932 10.1038/nature05541

[bib41] Vikhanskaya F, Lee MK, Mazzoletti M, Broggini M, Sabapathy K (2007) Cancer-derived p53 mutants suppress p53-target gene expression – potential mechanism for gain of function of mutant p53. Nucleic Acids Res 35: 2093–2104 17344317 10.1093/nar/gkm099 PMC1874625

[bib42] von Marschall Z, Cramer T, Hocker M, Burde R, Plath T, Schirner M, Heidenreich R, Breier G, Riecken EO, Wiedenmann B, Rosewicz S (2000) *De novo* expression of vascular endothelial growth factor in human pancreatic cancer: evidence for an autocrine mitogenic loop. Gastroenterology 119: 1358–1372 11054395 10.1053/gast.2000.19578

[bib43] Von Marschall Z, Scholz A, Stacker SA, Achen MG, Jackson DG, Alves F, Schirner M, Haberey M, Thierauch KH, Wiedenmann B, Rosewicz S (2005) Vascular endothelial growth factor-D induces lymphangiogenesis and lymphatic metastasis in models of ductal pancreatic cancer. Int J Oncol 27: 669–679 16077915

[bib44] Weidner N, Semple JP, Welch WR, Folkman J (1991) Tumor angiogenesis and metastasis – correlation in invasive breast carcinoma. N Engl J Med 324: 1–8 10.1056/NEJM199101033240101 1701519

[bib45] Xirodimas DP, Stephen CW, Lane DP (2001) Cocompartmentalization of p53 and Mdm2 is a major determinant for Mdm2-mediated degradation of p53. Exp Cell Res 270: 66–77 11597128 10.1006/excr.2001.5314

[bib46] Xue W, Zender L, Miething C, Dickins RA, Hernando E, Krizhanovsky V, Cordon-Cardo C, Lowe SW (2007) Senescence and tumour clearance is triggered by p53 restoration in murine liver carcinomas. Nature 445: 656–660 17251933 10.1038/nature05529 PMC4601097

